# Chloromethyl-triazole: a new motif for site-selective pseudo-acylation of proteins†
†Electronic supplementary information (ESI) available: Synthesis and characterisation of compounds **3b**, **4b**, and peptides **Pep1–3**. Peptide and protein alkylation procedures. See DOI: 10.1039/c6cc06801d. Primary data files can be found at http://dx.doi.org/10.7488/ds/1484



**DOI:** 10.1039/c6cc06801d

**Published:** 2016-09-13

**Authors:** Richard C. Brewster, Georgina C. Gavins, Barbara Günthardt, Sarah Farr, Kimberly M. Webb, Philipp Voigt, Alison N. Hulme

**Affiliations:** a EaSTCHEM School of Chemistry , The University of Edinburgh , Joseph Black Building , David Brewster Road , Edinburgh EH9 3FJ , UK . Email: Alison.Hulme@ed.ac.uk; b The Wellcome Trust Centre for Cell Biology , The University of Edinburgh , Michael Swann Building , Max Born Crescent , Edinburgh , EH9 3BF , UK

## Abstract

Rapid, site-selective modification of cysteine residues with chloromethyl-triazole derivatives generates pseudo-acyl sLys motifs, mimicking important post-translational modifications. Near-native biotinylation of peptide and protein substrates is shown to be site-selective and modified histone H4 retains functional activity.

## Abstract 2

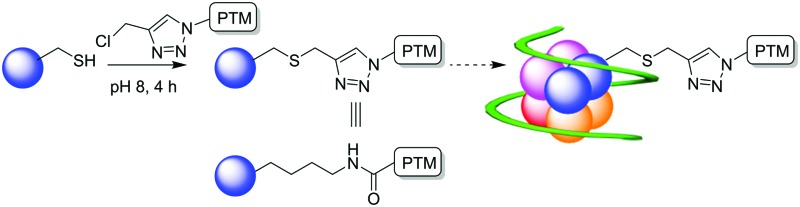
Chloromethyl triazoles are shown to be cysteine selective alkylation reagents for ‘near-native’ post-translational modification of protein and peptide substrates.

## 

Advances in techniques for the site-selective functionalization of proteins have revolutionised approaches to investigating the effects of post-translational modifications (PTMs) in recent years.^1^ Cysteine is an attractive target for chemical modification due to its relatively low abundance in proteins and the high reactivity of the sulfhydryl group compared to other amino acid side chain functional groups which ensures good selectivity.^2^ Indeed, in proteins such as histones where there are almost no native cysteine residues, a popular route to the systematic investigation of lysine modifications has been the selective modification of cysteine mutants to give near-native analogues of the parent PTM.^3^ This has been achieved either through the formation of a cleavable disulfide,^4^ or through *S*-alkylation to give thia-lysine derivatives (sLys),^5^ in which the only perturbation between the native lysine-containing histone and its cysteine-containing analogue is the switch of a CH_2_ for S. A range of amide derivatives of the side-chain of lysine are known as PTMs,^6^ with one example being the ‘native’ or endogenous biotinylation of proteins. The epigenetic role of biotin as a PTM of histones has been investigated recently.^7^ However, to date these studies have been hindered by the lack of a suitable reagent to chemically modify proteins with a near-native mimic of post-translational biotinylation, causing their conclusions to be questioned.^8^

sLys derivatives mimicking the different methylation states of lysine have been generated through the reaction of methylated chloro- or bromo-ethylamines.^5*c*^ Shokat *et al.* have shown that the alkylation reaction proceeds *via* an intermediate aziridine/aziridinium, which is subsequently ring-opened by the nucleophilic cysteine.^5*c*^ In contrast, in investigating the alkylation of cysteine to create acetyl lysine mimics, Cole *et al.* have shown that alkyl halide **1a** is virtually unreactive (Scheme 1).^5*b*^ Similarly, attempted ring-opening of a pre-formed acyl aziridine (not shown) has been shown to result in acyl transfer to the cysteine rather than formation of the acylated sLys derivative.^5*b*^ An alternative strategy, employing a thiol-ene reaction of *N*-vinyl acetamide **2a** has been used to generate acetyl lysine mimics on histones.^5*a*^ However, our attempted synthesis of the vinyl amide of biotin **2b** (either directly, or from bromo-ethyl derivative **1b**) was unsuccessful. We were thus drawn to an alternative approach in which a triazole mimic of the amide bond would be targeted,^9^ and the enhanced reactivity of the ‘pseudo-benzylic’ halide α to the triazole would give an efficient, high yielding alkylating reagent (**3b**, Scheme 1).

**Scheme 1:**
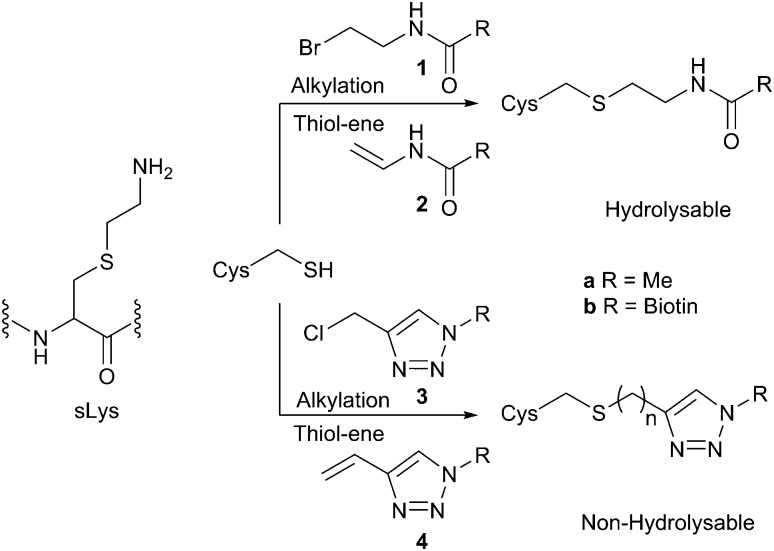
Routes to the formation of acyl, or pseudo-acyl sLys derivatives.

Synthesis of norbiotin azide **5** (Scheme 2) was achieved by a Curtius rearrangement of the carboxylic acid side chain on biotin **6**,^10^ followed by Boc deprotection and a diazo transfer reaction on the amine hydrochloride **7**, using imidazole-1-sulfonyl azide hydrochloride **8**.^11^ The CuAAC click reaction of azide **5** with homopropargyl bromide proceeded well under standard coupling conditions (65%).‡
‡CuAAC coupling conditions as in Scheme 2(d). The triazole adduct resulting from CuAAC click reaction of azide **5** with homopropargyl bromide was readily converted by elimination to the vinyltriazole biotin reagent **4b** (NaOH, EtOH, rt, 3 h, 79%). However, subsequent thiol-ene reaction with **Pep1** resulted in only moderate to poor conversion (<30%) under a range of conditions. However, in reactions with propargyl chloride and propargyl bromide only the azide starting material **5** was recovered under a range of CuAAC conditions. Gratifyingly, the desired product (which possesses both the optimum length of linkage for a near-native connection and enhanced reactivity) could be obtained by a CuAAC reaction of **5** with propargyl alcohol, and subsequent conversion to the chloromethyl-triazole **3b** using SOCl_2_. An alternative chlorination protocol,^12^ which employed treatment of the pseudo-benzylic intermediate alcohol with tosyl chloride gave chloromethyl-triazole **3b** in reduced yields. Attempts to synthesise the corresponding bromide from the intermediate alcohol using SOBr_2_ were very poor yielding, and the use of other brominating reagents was hindered by the poor solubility of the intermediate hydroxymethyl-triazole in organic solvents.

**Scheme 2:**
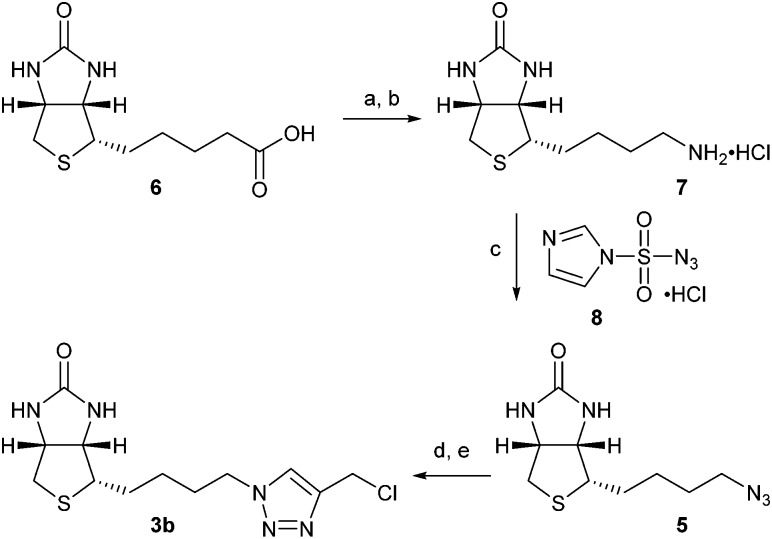
Synthesis of biotin alkylation reagent **3b**. (a) DPPA. TEA, ^*t*^BuOH, reflux, 18 h, 73%; (b) AcCl, EtOH, CHCl_3_, 4 h, 93%; (c) **8**, K_2_CO_3_, CuSO_4_·5H_2_O, MeOH, 75%; (d) propargyl alcohol, Cu(MeCN)_4_BF_4_, DMF, 20 h, 80%; (e) SOCl_2_, 0.5 h, 88%.

To probe the selectivity and reactivity of the new chloromethyl-triazole reagent, a series of peptide models was used. Monitoring the reaction by HPLC showed that alkylation of the cysteine in a short peptide sequence (**Pep1**: naphthalene-mPEG-GACR-OH) with **3b** under buffered conditions was complete in under 6 hours [**Pep1** (5.0 mM), **3b** (100 mM), DTT (20 mM), HEPES (pH 8.0), rt];§
§Minor modification of standard histone conditions, see: ref. 5*c*. no double alkylation product was detected by LC-MS (Fig. S1, ESI†). Using the same reaction conditions, further peptide sequences incorporating lysine and histidine residues (**Pep2**: FITC-βAla-GKAACF-NH_2_, **Pep3**: FITC-βAla-HGKAACF-NH_2_) showed that alkylation by **3b** was cysteine-selective (Fig. S2, ESI†). The rate of alkylation of **Pep1** by chloromethyl-triazole **3b** was then compared with that of other methylated chloro- (**9a–c**), or bromo-ethylamines (**10a**) typically used to create sLys methyl lysine mimics (Fig. 1).^3*a*^ As expected, the rate of reaction was observed to increase with increasing methylation of the amine (**9c** > **9b** > **9a**),^5*c*^ and the bromoethylamine **10a** was shown to react significantly faster than its chloroethylamine equivalent **9a**. However, chloromethyl-triazole **3b** reacts significantly faster than any of these reagents, with conversion levels of ∼80% reached in under 3 h. Whilst this rate of reaction is not as fast as that of a maleimide reagent (which is essentially instantaneous under equivalent conditions), it does provide a synthetically viable route for the selective and efficient alkylation of cysteine residues to give pseudo-acyl sLys derivatives.

**Fig. 1:**
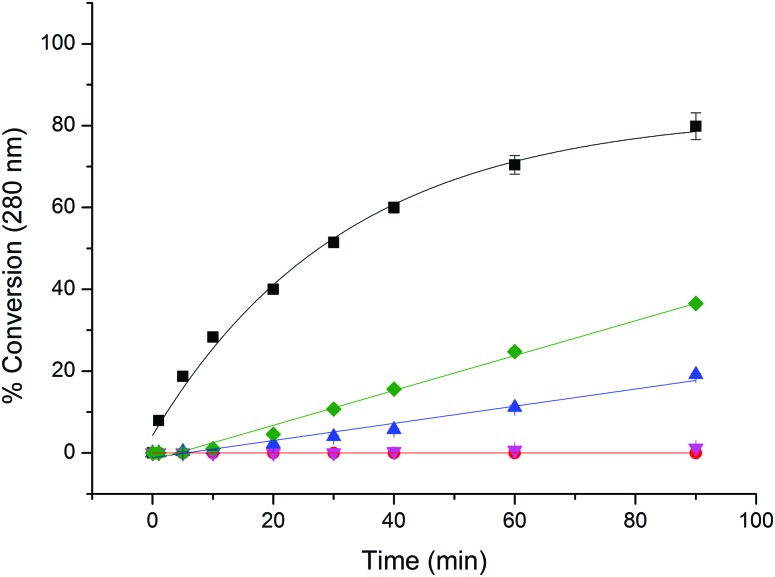
Relative rates of reaction of **Pep1** (naphthalene-mPEG-GACR-OH) with 2-chloroethylamine (**9a**, red), *N*-methyl 2-chloroethylamine (**9b**, magenta), *N*,*N*-dimethyl 2-chloroethylamine (**9c**, green), 2-bromoethylamine (**10a**, blue), and chloromethyl-triazole biotin (**3b**, black). Reactions conducted in the presence of 20 eq. alkylating reagent, 1 M HEPES pH 8, 5 eq. DTT, rt. Reactions were monitored by RP-HPLC (280 nm), and % conversion data represent the mean from 3 independent experiments. Error bars: ± standard deviation.

Methylation of lysine residues in histones is a well-documented epigenetic modification,^13^ and detailed protocols for the synthesis of near-native sLys analogues from the corresponding KxxC mutant histones have been published.^5^ Biotinylation of histone H4 (H4K12(bio)^14^ and H4K16(bio)^15^) has also been proposed to play an epigenetic role; with H4K16bio shown to affect chromatin condensation levels in studies conducted using non-native maleimide-PEG2-biotin reagents. Gratifyingly, treatment of the histone 4 mutant H4K12C with chloromethyl triazole **3b**, under standard histone alkylation conditions [protein (0.9 mM), **3b** (90 mM), DTT (20 mM), HEPES (pH 7.8), guanidine (4 M), rt]^16^ proceeded to completion in only 4 h. Purification using a size exclusion spin cartridge to desalt and remove excess alkylating reagent, and mass spectrometric analysis showed only the sLys biotin alkylated histone accompanied by very low levels of a double alkylation product (Fig. 2a). We have previously demonstrated that a range of non-hydrolysable triazole derivatives of biotin show very strong binding to avidin, with *K*_d_ values in the pM range.^17^ In this instance, functional activity of the H4K12C triazole biotin adduct was demonstrated by Western blot using an anti-biotin antibody (Fig. 2b), and the assembly of histone complexes incorporating site-selective biotinylation is currently under investigation.

**Fig. 2:**
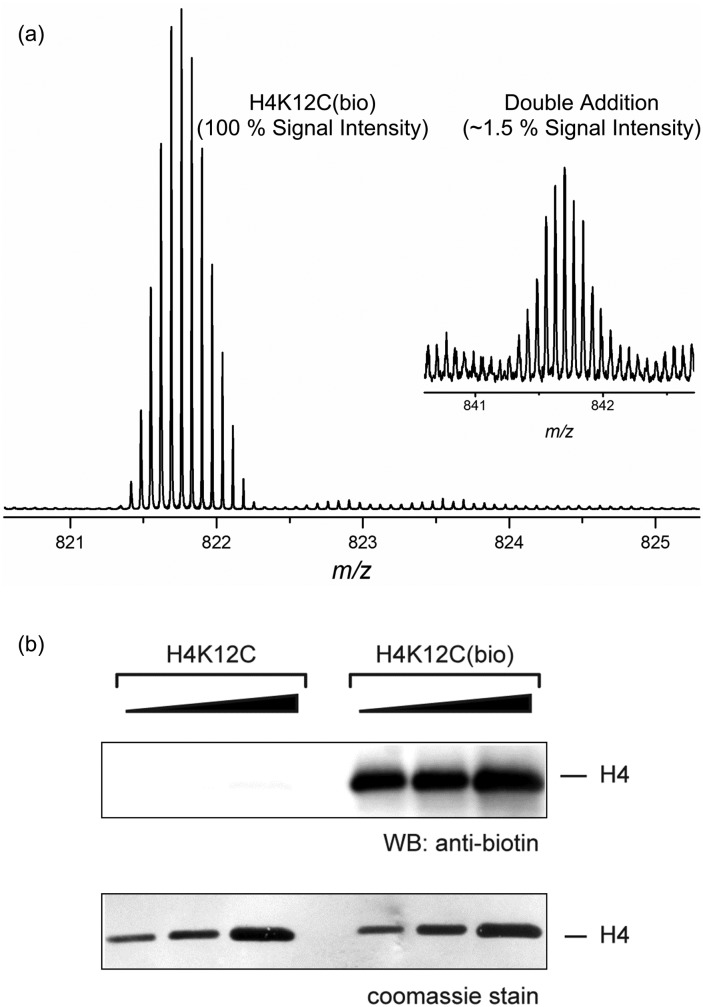
(a) Mass spectrometric analysis of the product of reaction of H4K12C with chloromethyl-triazole biotin reagent **3b**. (b) Western blot of the sLys biotin derivative of H4K12C using anti-biotin (Bethyl).

In summary, we have demonstrated that a chloromethyl-triazole motif can be used to introduce site selective, near-native mimics of amide-based PTMs into peptide and protein substrates. Using biotin chloromethyl-triazole **3b** as an example, the rate of cysteine alkylation was shown to be faster than that of commonly used *N*-methylated 2-haloethylamines; and functional activity of the resultant pseudo-acyl derivatives was confirmed by Western blot with anti-biotin. Due to the wide range of easily accessible functional azides and simple conversion to the corresponding chloromethyl-triazole, this motif could serve as a robust method for the rapid installation of PTM acetylation mimics into proteins. This chemical derivatisation approach complements acetylation techniques which rely on the genetic introduction of unnatural amino acids,^18^ which can be hampered by low protein expression levels. Ongoing efforts in our laboratories seek to expand this strategy to sugars, phosphates and fatty acids, and to exploit the high reactivity observed for chloromethyl-triazole based reagents in dual labelling studies.

This work was supported by the BBSRC (Grant Ref. BB/J01446X/1, EASTBIO studentship to RCB), the Wellcome Trust and the Royal Society (joint Grant Ref. 104175/Z/14/Z, Sir Henry Dale Fellowship to PV) and the Wellcome Trust through core funding to The Wellcome Trust Centre for Cell Biology (Grant Ref. 092076).

## References

[cit1] Wright T. H., Vallée M. R. J., Davis B. G. (2016). Angew. Chem., Int. Ed..

[cit2] Gunnoo S., Madder A. (2016). ChemBioChem.

[cit3] Müller M. M., Muir T. W. (2015). Chem. Rev..

[cit4] Chatterjee C., McGinty R. K., Fierz B., Muir T. W. (2010). Nat. Chem. Biol..

[cit5] Li F., Allahverdi A., Yang R., Lua G. B. J., Zhang X., Cao Y., Korolev N., Nardenskiöld L., Liu C. (2011). Angew. Chem., Int. Ed..

[cit6] Lin H., Su X., He B. (2012). ACS Chem. Biol..

[cit7] Kuroishi T., Rios-Avila L., Pestinger V., Wijeratne S. S. K., Zempleni J. (2011). Mol. Genet. Metab..

[cit8] Healy S., Perez-Cadahia B., Jia D., McDonald M. K., Davie J. R., Gravel R. A. (2009). Biochim. Biophys. Acta, Gene Regul. Mech..

[cit9] Kolb H. C., Sharpless B. K. (2003). Drug Discovery Today.

[cit10] Soares da Costa T. P., Tieu W., Yap M. Y., Zvarec O., Bell J. M., Turnidge J. D., Wallace J. C., Booker G. W., Wilce M. C. J., Abell A. D., Polyak S. W. (2012). ACS Med. Chem. Lett..

[cit11] Goddard-Borger E. D., Stick R. V. (2007). Org. Lett..

[cit12] Ding R., He Y., Wang X., Xu J., Chen Y., Feng M., Qi C. (2011). Molecules.

[cit13] Bannister A. J., Kouzarides T. (2011). Cell Res..

[cit14] Hassan Y. I., Zempleni J. (2006). J. Nutr..

[cit15] Singh M. P., Wijeratne S. S. K., Zempleni J. (2012). Arch. Biochem. Biophys..

[cit16] Simon M. D., in Current Protocols in Molecular Biology, ed. F. M. Ausubel, et al., John Wiley & Sons, Hoboken NJ, 2010, Unit 21.18.1–21.18.10. 10.1002/0471142727.mb2118s90 20373501

[cit17] Germeroth A. I., Hanna J. R., Karim R., Kundel F., Lowther J., Neate P. G. N., Blackburn E. A., Wear M. A., Campopiano D. J., Hulme A. N. (2013). Org. Biomol. Chem..

[cit18] Arbely E., Natan E., Brandt T., Allen M. D., Veprintsev D. B., Robinson C. V., Chin J. W., Joerger A. C., Fersht A. R. (2011). Proc. Natl. Acad. Sci. U. S. A..

