# Supplementary material for: Chloromethyl-triazole: a new motif for site-selective pseudo-acylation of proteins
Source: Chem Commun (Camb). 2016 Sep 13;52(82):12230–2. doi: 10.1039/c6cc06801d (PMC5656099; doi:10.1039/c6cc06801d)
Supplement: Supplementary file 1 [file CC-052-C6CC06801D-s001.pdf]

Electronic Supplementary Material (ESI) for ChemComm.
This journal is © The Royal Society of Chemistry 2016

Chloromethyl-triazole: A new motif for site-selective pseudo-acylation of proteins

Richard C. Brewster,[a] Georgina C. Gavins,[a] Barbara Günthardt,[a] Sarah Farr,[a] Kimberly M. Webb,[b] Philipp Voigt[b] and Alison N. Hulme*,[a]

[a.] *EaSTCHEM School of Chemistry, The University of Edinburgh, Joseph Black Building, David Brewster Road, Edinburgh EH9 3FJ, UK.*
[b.] *The Wellcome Trust Centre for Cell Biology, The University of Edinburgh, Michael Swann Building, Max Born Crescent, Edinburgh, EH9 3BF, UK.*

Page **Contents**

Page	Contents
S1	General Methods
S3	Synthesis
S6	Procedures and Methods for Solid Phase Peptide Synthesis
S7	Peptide Alkylation Procedures (inc. Figures S1 & S2)
S9	Histone Expression and Western Blots
S9	Protein Alkylation Procedure
S9	References
S10	^{1}H and ^{13}C NMR spectra

1.1 General Methods (Synthetic)

All non-aqueous reactions were carried out under an atmosphere of nitrogen or argon using oven-dried glassware that was cooled in a desiccator prior to use. Unless otherwise noted, starting materials and reagents were obtained from commercial suppliers and were used without further purification. Triethylamine and ^{t}BuOH were distilled from calcium hydride and stored over activated 4 Å molecular sieves under an argon atmosphere. Anhydrous DMF was purchased from Acros Organics.

Nuclear magnetic resonance (**NMR**) spectra were recorded at ambient temperature (298 K, unless otherwise stated) on a Bruker AVA400, AVA500 or AVA600 spectrometer running at 400, 500, or 600 MHz (^{1}H spectra) or 101, 126, 151 Hz (^{13}C spectra, respectively. Chemical shifts (δ values) are reported in parts-per-million (ppm) relative to tetramethylsilane (^{1}H and ^{13}C spectra; $\delta_{TMS} = 0$) and are calibrated to the residual solvent peak. ^{1}H NMR data are reported as follows: chemical shift, relative intensity, multiplicity (s = singlet, d = doublet, t = triplet, q = quartet, qn = quintet, m = multiplet, br = broad), coupling constants (*J* value, Hz), and interpretation. ^{13}C NMR data are reported as follows: chemical shift, relative intensity and assignment (C = quaternary, CH = methane, CH_2 = methylene, CH_3 = methyl).

Infra-red spectra (**IR**) were recorded neat on Shimadzu IRAffinity-1. The value of peaks at maximum absorbance (ν_{max}) are quoted in wavenumbers (cm^{-1}).

Optical rotations were obtained at ambient temperature with a cell of 1 dm length using an AA series polAAr 20 spectrometer operating at a wavelength of 589 nm (sodium D-line). Concentrations (*c*) for the derived specific rotations (**[α]$_D$**) are expressed in g/100 mL.

Mass spectra were obtained by electrospray (ESI) on a Bruker 12 T SolariX or Bruker microTOF II mass spectrometer. Mass-to-charge ratios (***m/z***) of all parent (molecular) ions ($[M]^{+/-}$) and their intensities are reported, followed by (major) fragment or adduct ions and their intensities.

Melting points (**mp**) were determined on a Gallenkamp Electrothermal Melting Point apparatus and are uncorrected the temperature range and whether the substance undergoes decomposition (dec.) over this range is reported.

R_f values (**R_f**) were recorded using Merck Silicagel 60 F_{254} aluminium backed plates. Flash chromatography was carried out using Merck Kieselgel 60 (Merck 9385) under positive pressure. Eluent compositions are quoted as v/v ratios.

1.2 General Methods (HPLC)

RP-HPLC analysis was performed using either a Waters Acquity UPLC PDA with a 100 x 2.4 mm BEH C18 1.7 µm column (System 1) or a Waters 600E pump, a Waters 717plus autosampler and a Waters 996 PDA equipped with a Phenomenex Luna C18(2), 5 µm, 250 x 4.6 mm column (System 2).

HPLC Method 1: System 2, H_2O (+0.1 %v/v TFA)/MeCN (+0.1 %v/v TFA), flow rate 1 mL min^{-1} at ambient temperature.

Time (min)	% H_2O (+0.1 % v/v TFA)	% MeCN (+0.1 % v/v TFA)
0	95	5
5	95	5
35	5	95
45	5	95
50	95	5

HPLC Method 2: System 1, H_2O (+0.1 %v/v TFA)/MeCN, flow rate 0.5 mL min^{-1} at 40 °C.

Time (min)	% H_2O (+0.1 % v/v TFA)	% MeCN
0	95	5
1	95	5
6	60	40
6.5	5	95
7.5	5	95
8	95	5

LC-MS Method 1: System 1, H_2O (+0.1 %v/v HCO_2H)/MeCN (+0.1 %v/v HCO_2H), flow rate 0.4 mL min^{-1} at 40 °C.

Time (min)	% H_2O (+0.1 % v/v HCO_2H)	% MeCN (+0.1 % v/v HCO_2H)
0	95	5
5	60	40
5.5	5	95
7	5	95
7.5	95	5

Semi-preparative RP-HPLC was performed by isocratic elution using a Waters 600E pump, a Waters 486 tuneable absorbance detector equipped with a Phenomenex Luna C18(2), 5 µm, 250 x 21.2 mm column at a flow rate of 21.2 mL min^{-1}.

Synthesis

(3*aS*,4*S*,6*aR*)-4-(4-*tert*-Butoxycarbonylaminobut-1-yl)-hexahydro-1*H*-thieno[3,4-d]imidazolidin-2-one, S1

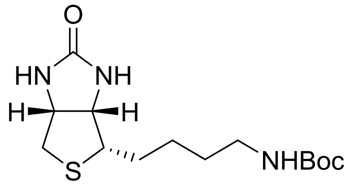

d-Biotin (1.22 g, 5.00 mmol) was mixed with anhydrous ^{t}BuOH (50 mL, distilled over CaH_2), triethylamine (0.77 mL, 5.50 mmol) and diphenylphosphoryl azide (1.19 mL, 5.50 mmol) under an argon atmosphere and the solution heated to reflux for 18 h. The reaction was concentrated *in vacuo* and purified by column chromatography (DCM:MeOH 19:1) yielding **S1** as a colourless solid (1.00 g, 70 %). $\mathbf{R_f}$ (19:1 DCM:MeOH) = 0.15; **mp** 162-164 °C; **[α]$_D$** = 145.4 (c 0.09, EtOH); **IR** (neat, cm^{-1}) 1681 (str, C=O), 1672 (str, C=O); **^{1}H NMR** δ (500 MHz, DMSO) 6.76 (1H, t, *J* = 5.1 Hz, $CH_2N\mathit{H}$Boc), 6.41 (1H, s, SCHCHN*H*), 6.34 (1H, s, $SCH_2CHN\mathit{H}$), 4.30 (1H, br dd, *J* = 7.5, 5.0 Hz, $SCH_2C\mathit{H}$), 4.13 (1H, ddd, *J* = 7.5, 4.6, 1.9 Hz, SCHC*H*), 3.09 (1H, ddd, *J* = 8.0, 6.6, 4.6 Hz, SC*H*), 2.90 (2H, q, *J* = 6.6 Hz, $C\mathit{H_2}$NHBoc), 2.82 (1H, dd, *J* = 12.4, 5.0 Hz, $SC\mathit{H_a}H_b$), 2.57 (1H, d, *J* = 12.4 Hz, $SCH_a\mathit{H_b}$), 1.68 – 1.54 (1H, m, $SCHC\mathit{H_c}H_d$), 1.52 – 1.21 (5H, m, $SCHCH_c\mathit{H_d}$, 2 × $C\mathit{H_2}$), 1.37 (9H, s, $(C\mathit{H_3})_3$); **^{13}C NMR** δ (126 MHz, DMSO) 162.7 (C), 155.6 (C), 77.3 (C), 60.9 (CH), 59.2 (CH), 55.5 (CH), 40.3* (CH_2), 40.1* (CH_2), 29.5 (CH_2), 28.3 (3 × CH_3), 28.0 (CH_2), 25.8 (CH_2). ***m/z*** (ESI+, MeCN) 653 ($[2M+Na]^+$, 27%) 338 ($[M+Na]^+$, 100) 316 ($[M+H]^+$, 4), 282 (79); **HRMS** $[M+H]^+$ found 316.1692, $C_{14}H_{26}N_3O_3S$ requires 316.1689. *Obscured by solvent peak – observed in HSQC.

(3*aS*,4*S*,6*aR*)-4-(4-Aminobut-1-yl)-hexahydro-1*H*-thieno[3,4-d]imidazolidin-2-one hydrochloride, 7

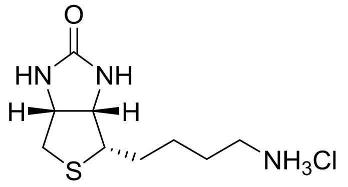

EtOH (1.31 mL, 22.4 mmol) was mixed with $CHCl_3$ (15 mL) at 0 °C. Acetyl chloride (1.59 mL, 22.4 mmol) was mixed with $CHCl_3$ and added to the EtOH solution. Boc protected norbiotinamine **S1** (1.00 g, 3.20 mmol) was partially dissolved in $CHCl_3$ (5 mL) at 0 °C and the acetyl chloride solution added to it over 5 min. The solution was warmed to rt and left to stir for 5 h after which time a solid had precipitated. The solid was filtered, washed with $CHCl_3$ (10 mL) and dried under vacuum yielding **7** as a colourless solid (760 mg, 95 %). **mp** 95-97 °C; **[α]$_D$** = 100.0 (c 0.12, EtOH); **IR** (neat, cm^{-1}) 3000 (br, NH), 1685 (str, C=O), 1661 (str, C=O); **^{1}H NMR** δ (500 MHz, CD_3OD) 4.64 (1H, ddd, *J* = 8.0, 4.3, 0.6 Hz, $SCH_2C\mathit{H}$), 4.46 (1H, dd, *J* = 8.0, 4.5 Hz, SCHC*H*), 3.32 – 3.28 (1H, m, SC*H*), 3.00 (1H, dd, *J* = 13.0, 5.0 Hz, $SC\mathit{H_a}H_b$), 2.95 (2H, t, *J* = 7.6 Hz, $C\mathit{H_2}NH_3Cl$), 2.79 (1H, d, *J* = 13.0 Hz, $SCH_a\mathit{H_b}$), 1.88 – 1.48 (6H, m, 3 × $C\mathit{H_2}$); **^{13}C NMR** δ (126 MHz, CD_3OD) 165.8 (C), 64.3 (CH), 62.7 (CH), 56.7 (CH), 40.7 (CH_2), 40.6 (CH_2), 29.3 (CH_2), 28.5 (CH_2), 27.1 (CH_2); ***m/z*** (ESI+, MeCN) 216 ($[M+H]^+$, 100), 199 ($[M-NH_2]^+$, 79); **HRMS** $[M+H]^+$ found 216.1168, $C_9H_{18}N_3OS$ requires 216.1165.

(3*aS*,4*S*,6*aR*)-4-(4-Azidobut-1-yl)-hexahydro-1*H*-thieno[3,4-*d*]imidazolidin-2-one, 5

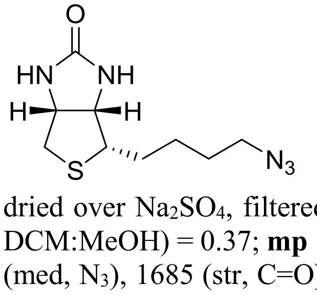

Imidazole-1-sulfonyl azide hydrochloride (1.1 g, 5.2 mmol) was added to norbiotinamine hydrochloride **7** (1.1 g, 4.4 mmol), K_2CO_3 (2.1 g, 15 mmol) and $CuSO_4•5H_2O$ (11 mg, 0.1 mmol) in MeOH (25 mL) and the mixture was stirred at rt for 18 h. The mixture was concentrated *in vacuo*, diluted with H_2O (60 mL), acidified with conc. HCl and extracted with $CHCl_3$ (3 x 40 mL). The combined organic layers were washed with brine (2 × 40 mL), dried over Na_2SO_4, filtered and concentrated to afford **5** as a colourless solid (0.78 g, 74%): $\mathbf{R_f}$ (9:1 DCM:MeOH) = 0.37; **mp** 140-142 °C; **[α]$_D$** = 77.6 (c 1.02, EtOH); **IR** (neat, cm^{-1}) 3250 (w, NH), 2091 (med, N_3), 1685 (str, C=O); **^{1}H NMR** δ (500 MHz, d_6-DMSO) 6.43 (1H, s, SCHCHN*H*), 6.35 (1H, s, $SCH_2CHN\mathit{H}$), 4.31 (1H, ddt, *J* = 7.6, 5.1, 1.2 Hz, $SCH_2C\mathit{H}$), 4.14 (1H, ddd, *J* = 7.6, 4.5, 1.8 Hz, SCHC*H*), 3.32 (2H, t, *J* = 6.9 Hz, $C\mathit{H_2}N_3$), 3.11 (1H, ddd, *J* = 8.4, 6.3, 4.5 Hz, SC*H*), 2.83 (1H, dd, *J* = 12.4, 5.1 Hz, $SC\mathit{H_a}H_b$), 2.58 (1H, d, *J* = 12.4 Hz, $SCH_a\mathit{H_b}$), 1.69 – 1.29 (6H, m, 3 × $C\mathit{H_2}$); **^{13}C NMR** δ (126 MHz, d_6-DMSO) 162.7 (C), 61.0 (CH), 59.2 (CH), 55.3 (CH), 50.5 (CH_2), 40.3* (CH_2), 28.3

(CH_2), 27.8 (CH_2), 25.8 (CH_2); ***m/z*** (ESI+, MeCN) 505 ($[2M+Na]^+$, 14%), 483 ($[2M+H]^+$, 36), 264 ($[M+Na]^+$, 63), 242 ($[M+H]^+$, 100), 197 (39); **HRMS** $[M+Na]^+$ found 264.0892, $C_9H_{15}N_5OSNa$ requires 264.0890. *Obscured by solvent peak – observed in HSQC.

(3*aS*,4*S*,6*aR*)-4-{4-[4-(Hydroxymethyl)-1*H*-1,2,3-triazol-1-yl]but-1-yl}-hexahydro-1*H*-thieno[3,4-*d*]imidazolidin-2-one, S2

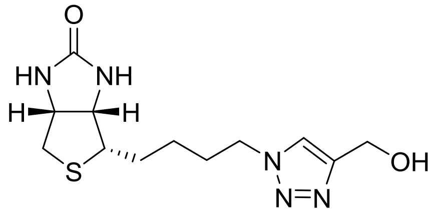

To a solution of norbiotin azide **5** (150 mg, 0.60 mmol) in degassed DMF (5 mL) was added $Cu(MeCN)_4BF_4$ (16 mg, 10 mol%), and propargyl alcohol (93 mg, 97 µL, 1.7 mmol). The reaction was stirred in the dark at rt for 20 h. Celite (2 g) was added to the reaction mixture and the solvent was removed *in vacuo*. Dry-loading the Celite layer allowed the product to be purified by column chromatography (gradient elution, 9:1 to 8:2 DCM:MeOH) to yield **S2** as a colourless solid (154 mg, 80% yield). $\mathbf{R_f}$ (17:3 DCM:MeOH) = 0.30; **mp** 160-162 °C; $\mathbf{[\alpha]_D}$ = 90.9 (c 0.11, EtOH); **IR** (neat, cm^{-1}) 3240 (br, OH), 1690 (str, C=O); $\mathbf{^1H}$ **NMR** δ (600 MHz, d_6-DMSO) 7.94 (1H, s, NC*H*), 6.42 (1H, s, SCHCHN*H*), 6.35 (1H, s, $SCHCH_2N$*H*), 5.13 (1H, br s, CH_2O*H*), 4.51 (2H, s, C*H_2*OH), 4.33 (2H, t, J = 7.1 Hz, CH_2C*H_2*N), 4.34 – 4.28 (1H, m, SCH_2C*H*), 4.13 (1H, ddd, J = 7.7, 4.5, 1.7, SCHC*H*), 3.09 (1H, ddd, J = 8.4, 6.4, 4.5 Hz, SC*H*), 2.82 (1H, dd, J = 12.4, 5.1 Hz, SCH_aH_b), 2.58 (1H, d, J = 12.4 Hz, SCH_aH_b), 1.87 – 1.78 (2H, m, CH_2CH_2N), 1.68 – 1.62 (1H, m, $SCHCH_cH_d$), 1.54 – 1.48 (1H, m, $SCHCH_cH_d$), 1.37 – 1.25 (2H, m, $CH_2CH_2CH_2N$); $\mathbf{^{13}C}$ **NMR** δ (126 MHz, d_6-DMSO) 162.6 (C), 147.9 (C), 122.5 (CH), 60.9 (CH), 59.1 (CH), 55.2 (CH), 55.1 (CH_2), 49.0 (CH_2), 40.3* (CH_2), 29.8 (CH_2), 27.7 (CH_2), 25.4 (CH_2); ***m/z*** (ESI+, MeCN) 320 ($[M+Na]^+$, 43%), 298 ($[M+H]^+$, 100), 252 (87), 199 (18), 197 (17); **HRMS** $[M+H]^+$ found 298.1329, $C_{12}H_{20}N_5O_2S$ requires 298.1332. *Obscured by solvent peak, observed in HSQC.

(3*aS*,4*S*,6*aR*)-4-{4-[4-(2-Bromoethyl)-1*H*-1,2,3-triazol-1-yl]but-1-yl}-hexahydro-1*H*-thieno[3,4-*d*]imidazolidin-2-one, S3

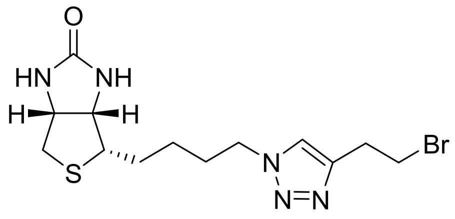

To a solution of norbiotin-azide **5** (55 mg, 0.23 mmol) in degassed, dry DMF (5 mL) was added $Cu(MeCN)_4BF_4$ (7.2 mg, 10 mol %) and 4-bromo-1-butyne (83 mg, 59 µl, 0.50 mmol). The reaction was stirred in the dark at rt for 4 h. Celite (1 g) was added to the reaction mixture and the solvent removed *in vacuo*. Dry-loading the Celite layer allowed the product to be purified by column chromatography (gradient elution, 19:1 to 9:1 DCM:MeOH) to yield **S3** as a colourless solid (55 mg, 0.15 mmol, 65% yield). $\mathbf{R_f}$ (9:1 DCM:MeOH) = 0.35; **mp** 160 °C (dec); $\mathbf{[\alpha]_D}$ = 96.1 (c 0.10, EtOH); **IR** (neat, cm^{-1}) 3225 (w, NH), 1690 (str, C=O); $\mathbf{^1H}$ **NMR** δ (600 MHz, d_6-DMSO) 7.96 (1H, s, NC*H*), 6.42 (1H, s, SCHCHN*H*), 6.35 (1H, s, $SCHCH_2N$*H*), 4.32 (2H, t, J = 7.2 Hz, CH_2C*H_2*N), 4.33 – 4.28 (1H, m, SCH_2C*H*), 4.12 (1H, ddd, J = 7.6, 4.5, 1.8 Hz, SCHC*H*), 3.73 (2H, t, J = 6.9 Hz, CH_2C*H_2*Br), 3.18 (2H, t, J = 6.9 Hz, CH_2CH_2Br), 3.08 (1H, ddd, J = 8.3, 6.4, 4.5 Hz, SC*H*), 2.82 (1H, dd, J = 12.4, 5.1 Hz, SCH_aH_b), 2.57 (1H, d, J = 12.4 Hz, SCH_aH_b), 1.89 – 1.76 (2H, m, CH_2CH_2N), 1.70 – 1.59 (1H, m, $SCHCH_cH_d$), 1.55 – 1.45 (1H, m, $SCHCH_cH_d$), 1.38 – 1.22 (2H, m, $CH_2CH_2CH_2N$); $\mathbf{^{13}C}$ **NMR** δ (126 MHz, d_6-DMSO) 162.6 (C), 144.0 (C), 122.5 (CH), 60.9 (CH), 59.2 (CH), 55.2 (CH), 49.0 (CH_2), 40.3* (CH_2), 32.8 (CH_2), 29.7 (CH_2), 29.0 (CH_2), 27.7 (CH_2), 25.4 (CH_2); ***m/z*** (ESI+, MeCN) 398 ($[^{81}BrM+Na]^+$, 11%), 396 ($[^{79}BrM+Na]^+$, 11), 376 ($[^{81}BrM+H]^+$, 100), 374 ($[^{79}BrM+H]^+$, 92) 199 (12); **HRMS** (ESI+, MeCN) $[^{79}BrM+H]^+$ found 374.0648, $C_{13}H_{21}{}^{79}BrN_5OS$ requires 374.0645. *Obscured by solvent peak, observed in HSQC.

(3*aS*,4*S*,6*aR*)-4-{4-[4-(Chloromethyl)-1*H*-1,2,3-triazol-1-yl]but-1-yl}-hexahydro-1*H*-thieno[3,4-*d*]imidazolidin-2-one, 3b

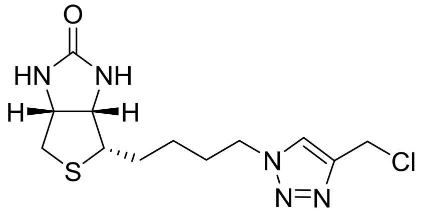

Alcohol **S2** (50 mg, 0.17 mmol) was added to $SOCl_2$ (2 mL) at 0 °C and stirred at rt for 30 min. The $SOCl_2$ was removed under a stream of N_2 and the resulting dark yellow oil was purified by column chromatography (9:1 DCM:MeOH) yielding **3b** as a colourless solid (49 mg, 88% yield). **R_f** (9:1 DCM:MeOH) = 0.34; **mp** 190 °C (dec); **[α]$_D$** = 96.1 (c 0.10, EtOH); **IR** (neat, cm^{-1}) 3225 (w, NH), 1690 (str, C=O); **^{1}H NMR** δ (600 MHz, CD_3OD) 8.04 (1H, s, NC*H*), 4.73 (2H, s, C*H$_2$Cl*), 4.49 (1H, ddd, *J* = 7.9, 5.0, 0.8 Hz, SCH_2CH), 4.46 – 4.40 (2H, td, *J* = 7.1, 0.8, C*H$_2$*N), 4.29 (1H, dd, *J* = 7.9, 4.5 Hz, SCHC*H*), 3.22 – 3.16 (1H, m, SC*H*), 2.93 (1H, dd, *J* = 12.7, 5.0 Hz, SCH_aH_b), 2.71 (1H, d, *J* = 12.7 Hz, SCH_aH_b), 2.04 – 1.91 (2H, m, CH_2CH_2N), 1.82 – 1.74 (1H, m, $SCHCH_cH_d$), 1.66 – 1.58 (1H, m, $SCHCH_cH_d$), 1.48 – 1.40 (2H, m, $CH_2CH_2CH_2N$); **^{13}C NMR** δ (126 MHz, d_6-DMSO) 162.6 (C), 143.3 (C), 124.0 (CH), 60.9 (CH), 59.2 (CH), 55.2 (CH), 49.2 (CH_2), 40.3* (CH_2), 36.6 (CH_2), 29.6 (CH_2), 27.7 (CH_2), 25.4 (CH_2); ***m/z*** (ESI+, MeCN) 318 ([^{37}ClM+H]$^+$, 38%), 316 ([^{35}ClM+H]$^+$, 100), 252 (68), 199 (34); **HRMS** (ESI+, MeCN) [^{35}ClM+H]$^+$ found 316.0988, $C_{12}H_{19}N_5OS^{35}Cl$ requires 316.0993. *Obscured by solvent peak, observed in HSQC.

(3a*S*,4*S*,6*aR*)-4-[4-(4-Ethenyl-1*H*-1,2,3-triazol-1-yl)but-1-yl]-hexahydro-1*H*-thieno[3,4-*d*]imidazolidin-2-one, 4b

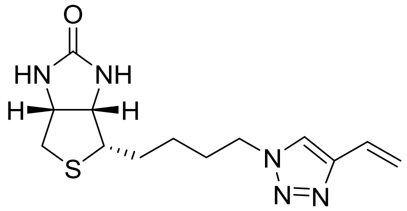

Bromide **S3** (50 mg, 0.13 mmol) was dissolved in EtOH (10 mL) and NaOH (10 mL; 1 M aq) and stirred at rt for 3 h. The reaction mixture was lyophilised and dissolved in 4:1 H_2O:MeCN (5 mL). The solution was acidified with TFA (10% v/v aq) and purified by RP HPLC [isocratic 80/20 H_2O (+0.1% v/v TFA)/MeCN (+0.1% v/v TFA), R_t = 12.2 min]. The solution was lyophilised to yield **4b** as a fluffy colourless powder (30 mg, 79% yield); **R_t** = 22.3 min (HPLC Method 1); **[α]$_D$** = 100.0 (c 0.04, EtOH); **IR** (neat, cm^{-1}) 1690 (str, C=O); **^{1}H NMR** δ (500 MHz, CD_3OD) 8.02 (1H, s, NC*H*), 6.70 (1H, dd, *J* = 17.8, 11.2 Hz, CC*H*CH_2), 5.89 (1H, dd, *J* = 17.8, 1.2 Hz, $CHCH_{trans}H_{cis}$), 5.35 (1H, dd, *J* = 11.2, 1.2 Hz, $CHCH_{trans}H_{cis}$), 4.49 (1H, dd, *J* = 7.9, 4.7 Hz, SCH_2CH), 4.41 (2H, t, *J* = 7.1 Hz, C*H$_2$*N), 4.30 (1H, dd, *J* = 7.9, 4.5 Hz, SCHC*H*), 3.19 (1H, br dt, *J* = 9.9, 5.5 Hz, SC*H*), 2.93 (1H, dd, *J* = 12.7, 5.0 Hz, SCH_aH_b), 2.71 (1H, d, *J* = 12.7 Hz, SCH_aH_b), 2.04 – 1.89 (2H, m, CH_2CH_2N), 1.84 – 1.72 (1H, m, $SCHCH_cH_d$), 1.70 – 1.57 (1H, m, $SCHCH_cH_d$), 1.50 – 1.38 (2H, m, $CH_2CH_2CH_2N$); **^{13}C NMR** δ (126 MHz, CD_3OD) 166.1 (C), 147.4 (C), 126.5 (C), 122.7 (C), 116.7 (C), 63.4 (CH), 61.6 (CH), 56.8 (CH), 51.1 (CH_2), 41.0 (CH_2), 31.1 (CH_2), 29.2 (CH_2), 27.0 (CH_2); ***m/z*** (ESI+, MeCN) 316 ([M+Na]$^+$, 33%), 294 ([M+H]$^+$, 100), 266 (29), 199 (16); **HRMS (**ESI+, MeCN) [M+H]$^+$ found 294.1390, $C_{13}H_{20}N_5OS$ requires 294.1383.

Procedures and Methods for Solid Phase Peptide Synthesis

For peptide synthesis, all amino acids and resins were purchased from either Novasyn (Merck) or Sigma-Aldrich and Fmoc-mPEG-OH was purchased from IRIS Biotech. All amino acids were *N*-Fmoc protected and side chains were protected with Boc (Lys); Trt (His, Cys); Pbf (Arg). Peptides were synthesised on Rink Amide AM resin with a loading capacity of 0.69 mmol g^{-1} or 2-chlorotrityl chloride resin with a loading of 1.0-1.5 mmol g^{-1}. Synthesis of peptides was performed manually using SPE tube and draining to a vacuum box. Agitation of the resin was carried out on a Stuart SB2 tube spinner at 20 rpm. Monitoring of coupling steps was performed using tandem chloranil and TNBS testing.

Method A: Resin Swelling
The required quantity of resin was weighed directly into an appropriate sized SPE tube equipped with filter and spigot. This was charged with DCM which was slowly allowed to drain from the tube. After several washes of the resin in this fashion the resin was suspended in DCM and left for 30 mins.

Method B: Deprotection of *N*-Fmoc Protecting Groups
N-Fmoc protecting groups were removed by the addition of piperidine (20% in DMF) to the resin and agitation (10 min), followed by washing of the resin (2 × DMF, 2 × DCM, 1 × DMF). This process was repeated a second time to ensure deprotection.

Method C: Tandem TNBS and chloranil testing
The TNBS test was employed to ensure deprotection and/or coupling of an amino acid residue. A small number of resin beads were placed into an Eppendorf and suspended in a small amount of DMF. To this was added several drops of DIPEA (10% in DMF) and 1 drop of TNBS solution (5% aq). The Eppendorf was then agitated (10 min) and the beads inspected, colourless beads indicated a successful coupling while bright red beads indicated a successful deprotection.

The chloranil test was run in parallel. A small number of resin beads were placed into an Eppendorf and suspended in a small amount of DMF. To this was added several drops of freshly made chloranil (2% in DMF) and several drops of acetaldehyde (2% in DMF). Immediate colour change indicated the successful deprotection of a secondary amine residue. The Eppendorf was then agitated (10 min) and the beads inspected, colourless beads indicated a successful coupling while blue beads indicated a successful primary amine deprotection.

Method D: Coupling of Amino acids
The desired amino acid (3.0 eq.) and OxymaPure® (3.0 eq.) were premixed in a small amount of DMF (5 min). To this was added DIC (3.0 eq.), the mixture was vortexed (1 min) and added to the resin. The resin was then agitated for 45 min and subsequently washed (2 × DMF, 2 × DCM, 1 × DMF).
Coupling efficiency was tested (Method C): a negative result indicating complete coupling and a positive result requiring a repeat coupling of the amino acid. Following complete coupling, *N*-Fmoc deprotection of was achieved *via* Method B.

Method E: Resin Storage
The peptide was washed (4 × MeOH) and suspended in MeOH for 10 min to shrink the resin. After drainage the resin, tube and lid was dried using Et_2O, tightly sealed and placed in a -20 °C freezer.

Method F: Peptide Capping
2-Napthoic acid was coupled using standard coupling conditions (Method D); coupling was confirmed using the TNBS test.
FITC (5 eq) was added in minimal volume of DIPEA and agitated for 16 h. The resin was washed sequentially with DCM and DMF until the eluent turned colourless. Coupling was confirmed by performing a mini cleave and analysis by HPLC/MS.

Method G: Cleavage and Global Deprotection

The peptides were deprotected and cleaved from the resin by treatment with TFA:TIS:H_2O, (95:2.5:2.5) and agitation for 2 h. The resin was then drained into a centrifuge tube and the resin washed with TFA and the combined eluents concentrated under N_2. The resulting oil was then precipitated using cold ether, the solid recovered by centrifugation (3200×g, 2 min), the supernatant removed and the pellet rinsed a further 4 times with cold ether. The solid was then dissolved in H_2O (0.1% v/v TFA) and acetonitrile (0.1% v/v TFA) and lyophilised.

Method H: Peptide Purification

Initially the crude peptides were dissolved in H_2O (+0.1% v/v TFA):MeCN (+0.1% v/v TFA) and analysed by analytical HPLC using Method 1. From this an appropriate isocratic eluent was selected for semi-preparative scale HPLC where the bulk of the peptide was purified. Injections (2.0 mL) and fraction collection were performed manually, monitoring was provided by UV detection at 210 nm. Fractions were checked for purity of >90 % by HPLC, combined and lyophilized.

Pep1: Naphth-mPEG-GACR-OH. $\mathbf{R_t}$ = 23.3 min (HPLC Method 1); ***m/z*** (ESI+, MeCN) 727 ($[M+Na]^+$, 10%), 705 ($[M+H]^+$, 100); **HRMS** (ESI+, MeCN) $[M+H]^+$ found 705.3023, $C_{31}H_{45}N_8O_9S$ requires 705.3025.
Pep2: FITC-βAla-GKAACF-NH_2. $\mathbf{R_t}$ = 24.0 min (HPLC Method 1); ***m/z*** (ESI+, MeCN) 1077 ($[M+Na]^+$, 18%), 1055 ($[M+H]^+$, 100), 528 ($[2M+H]^+$, 90); **HRMS** (ESI+, MeCN) $[M+H]^+$ found 1055.3881, $C_{50}H_{59}N_{10}O_{12}S_2$ requires 1055.3750.
Pep3: FITC-βAla-HGKAACF-NH_2. $\mathbf{R_t}$ = 23.0 min (HPLC Method 1); ***m/z*** (ESI+, MeCN) 1214 ($[M+Na]^+$, 9%), 1192 ($[M+H]^+$, 100), 596 ($[2M+H]^+$, 55); **HRMS** (ESI+, MeCN) $[M+H]^+$ found 1192.4460, $C_{56}H_{66}N_{13}O_{13}S_2$ requires 1192.4339.

Alkylation of peptides

Alkylation procedure was adapted from Shokat *et al.*[1]

Reagents stock solutions are as follows: Peptide (1.0 M in HEPES 1.0 M, pH 8); Alkylating reagents: 2-chloroethylamine **9a** (1.0 M in H_2O); *N*-methyl 2-chloroethylamine **9b** (1.0 M in H_2O); *N,N*-dimethyl 2-chloroethylamine **9c** (1.0 M in H_2O); 2-bromoethylamine **10a** (1.0 M in H_2O); chloromethyl-triazole biotin **3b** (1.0 M DMSO). DTT (1.0 M in H_2O) was prepared directly before use.

DTT (5 μL, 5 eq.) was added to the peptide (100 μL) and the solution was incubated at 37 °C for 1 h. Buffer (75 μL, HEPES 1.0 M, pH 8) was added, followed by alkylating reagent (20 μL, 20 eq.). The solution was mixed thoroughly with a pipette giving final concentrations of peptide (5 mM), DTT (20 mM) and alkylating reagent (100 mM). Reactions were kept in the dark at rt.
Reactions were monitored by removing an aliquot (5 μL) quenching by the addition of H_2O (300 μL, 0.5 % v/v TFA) and analysis by HPLC Method 2 or LC-MS Method 1.

Peptide		Starting Material	**10a**	**9b**	**9c**	**3b**
**Pep 1**	R_t	4.38	3.05	3.89	3.91	4.61
	Mass	705.30	748.34	762.36	776.37	984.42
	Expected Mass	705.30	748.34	762.36	776.37	984.42
**Pep 2**	R_t	4.60	4.14	4.17	4.19	4.70
	Mass	1055.36	1098.41	1112.43	1126.44	1334.49
	Expected Mass	1055.37	1098.41	1112.4334	1126.4490	1334.49
**Pep 3**	R_t	4.27	3.83	3.85	3.86	4.43
	Mass	1192.42	1235.47	1249.49	1263.51	1471.54
	Expected Mass	1192.43	1235.47	1249.49	1263.51	1471.54

Table 1: LC/MS data for alkylation reactions, analysis by LCMS Method 1.

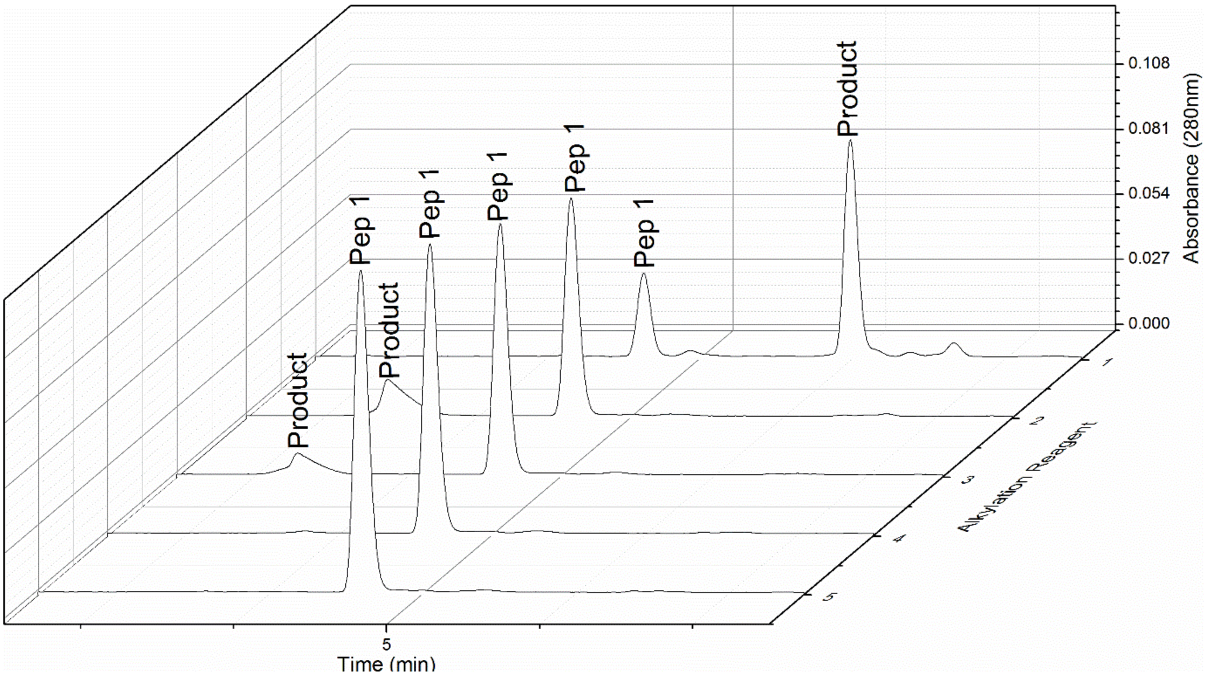

Figure S1: RP-HPLC (280 nm) trace of alkylation reactions on **Pep1** after 60 minutes. 1 = **3b**, 2 = **9c**, 3 = **10a**, 4 = **9b**, 5 = **9a**, reactions were monitored by HPLC Method 2.

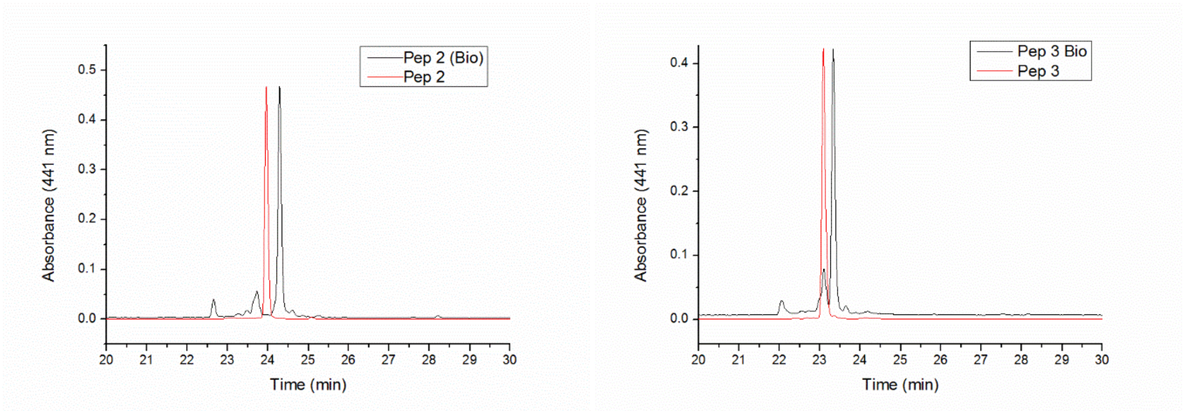

Figure S2: RP-HPLC (441 nm) trace of crude alkylation reactions on **Pep 2** (left) and **Pep 3** (right) with **3b** using HPLC method 1.

Histone Expression

Histone H4 from Xenopus laevis[2] with a lysine-to-cysteine mutation at position 12 (H4K12C) was expressed in *E. coli*, solubilized from inclusion bodies and purified by sequential anion and cation exchange chromatography on HiTrap Q and SP columns (GE). Fractions were analysed by SDS-PAGE and Coomassie staining. Histone-containing fractions were pooled, dialyzed against 5 mM 2-mercaptoethanol and lyophilized for storage and buffer exchange.

Alkylation of H4K12C

Alkylation Buffer: 4 M Guanidine hydrochloride, 1 M HEPES pH 7.8, 10 mM DL-methionine.

Alkylation was performed using minor modifications to the reported method.[3]

Lyophilised H4K12C (10 mg) was mixed in an Eppendorf tube with 890 µL of alkylation buffer and DTT (20 µL, 1 M in H_2O) and incubated at 37 °C for 1 h. **3b** (90 µL, 1 M in DMSO) was added and the solution was left in the dark at rt for 4 h. The reaction was quenched by addition of 2-mercaptoethanol (50 µL, neat) and the quenched reaction mixture was loaded onto a PD-10 column (GE), previously equilibrated with 2-mercaptoethanol (25 mL, 2 mM in H_2O). The vial was rinsed with 2-mercaptoethanol (2 × 725 µL, 2 mM in H_2O) and the washings loaded onto the column, bringing the total volume to 2.5 mL. 3.2 mL of the eluate was then collected, flash frozen in liquid N_2 and lyophilised yielding H4K12Cbio (6 mg) as a colourless solid.

Western Blotting

Presence of the biotin moiety in H4K12bio was verified by Western Blotting with an anti-biotin antibody (Bethyl, via Cambridge Bioscience).

References

1. M. D. Simon, F. Chu, L. R. Racki, C.C. de la Cruz, A. L. Burlingame, B. Panning, G. J. Narlikar and K. M. Shokat, *Cell*, 2007, **128**, 1003-1012.
2. Luger, K., Rechsteiner, T. J., Flaus, A. J., Waye, M. M. & Richmond, T. J. *J. Mol. Biol.* 1997, **272**, 301–311.
3. M. D. Simon, in *Current Protocols in Molecular Biology*, eds. F. M. Ausubel *et al.*, John Wiley & Sons, Hoboken NJ, 2010, Unit 21.18.1–21.18.10.

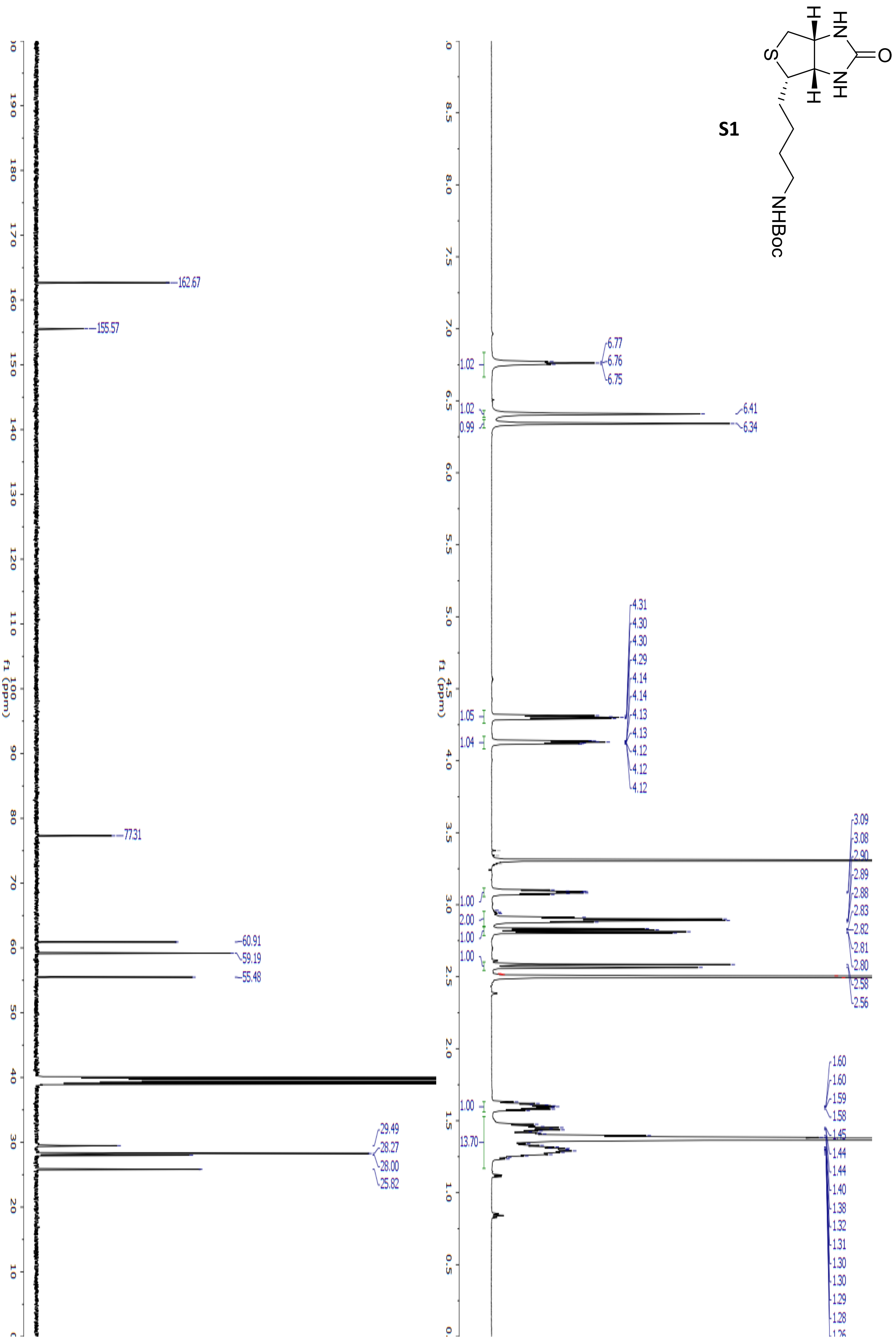

S1
NHBoc
f1 (ppm)
162.67
155.57
77.31
60.91
59.19
55.48
29.49
28.27
28.00
25.82
6.77
6.76
6.75
6.41
6.34
1.02
1.02
0.99
1.05
1.04
1.00
2.00
1.00
1.00
1.00
13.70

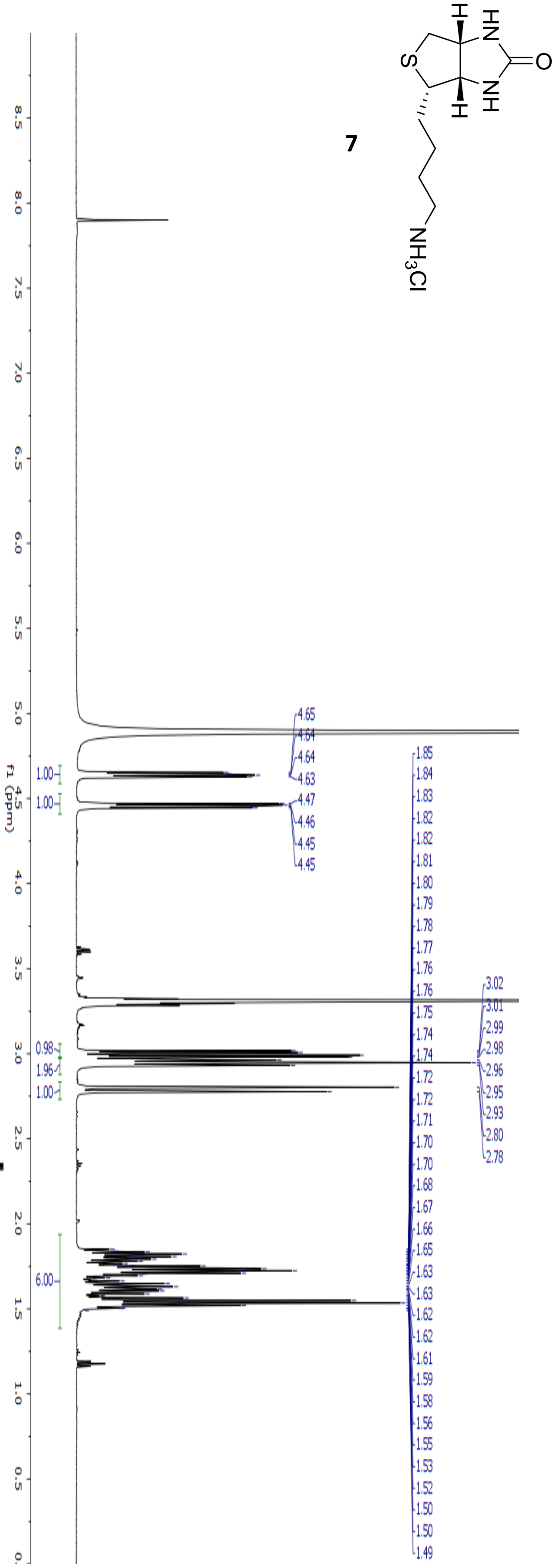

HN
NH
O
H
H
S
7
NH3Cl
4.65
4.64
4.64
4.63
4.47
4.46
4.45
4.45
1.85
1.84
1.83
1.82
1.82
1.81
1.80
1.79
1.78
1.77
1.76
1.76
1.75
1.74
1.74
1.72
1.72
1.71
1.70
1.70
1.68
1.67
1.66
1.65
1.63
1.63
1.62
1.62
1.61
1.59
1.58
1.56
1.55
1.53
1.52
1.50
1.50
1.49
3.02
3.01
2.99
2.98
2.96
2.95
2.93
2.80
2.78
1.00
1.00
0.98
1.96
1.00
6.00
f1 (ppm)

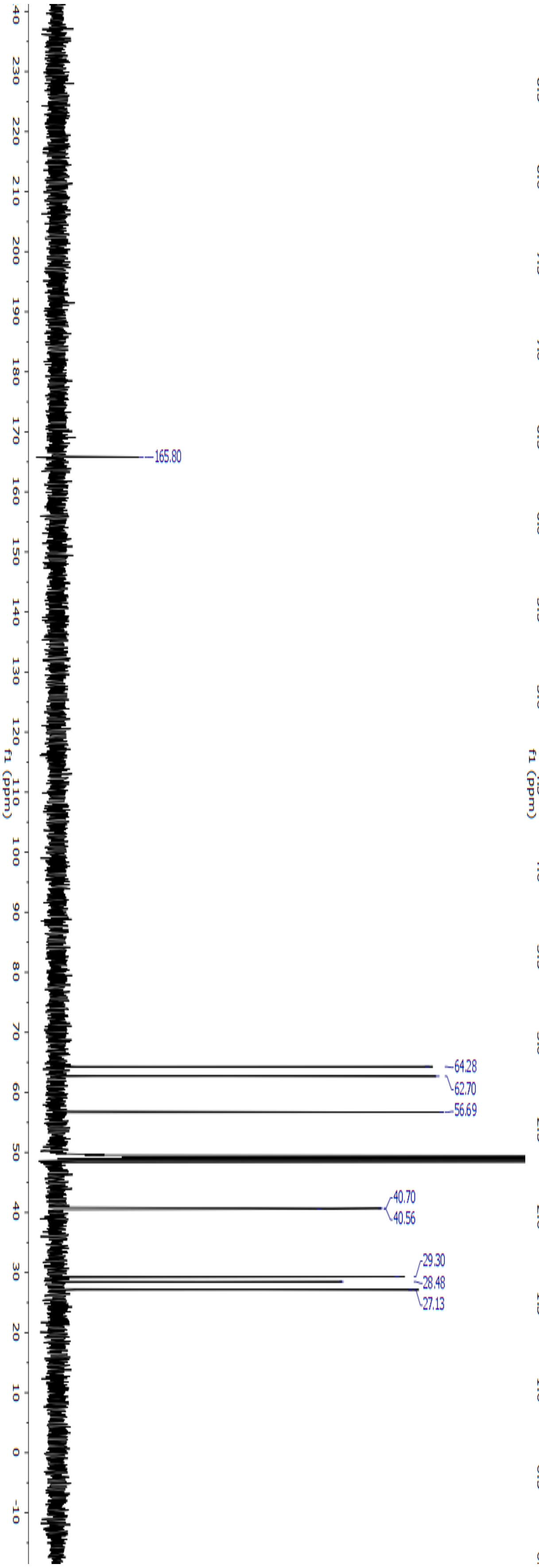

165.80
64.28
62.70
56.69
40.70
40.56
29.30
28.48
27.13
f1 (ppm)

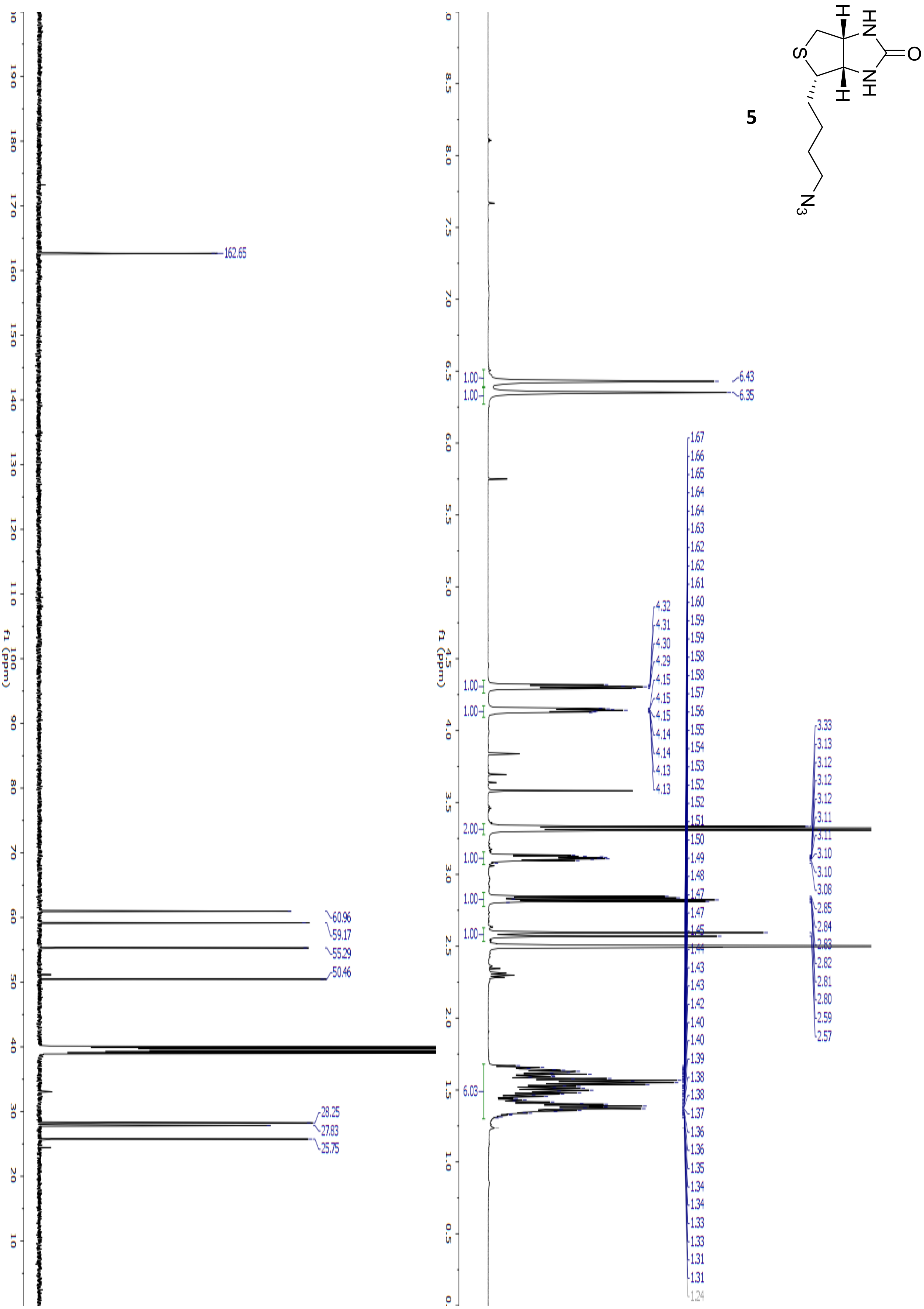
5
f1 (ppm)
162.65
60.96
59.17
55.29
50.46
28.25
27.83
25.75
6.43
6.35

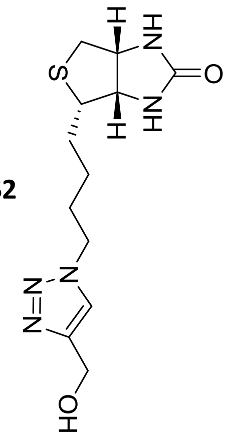

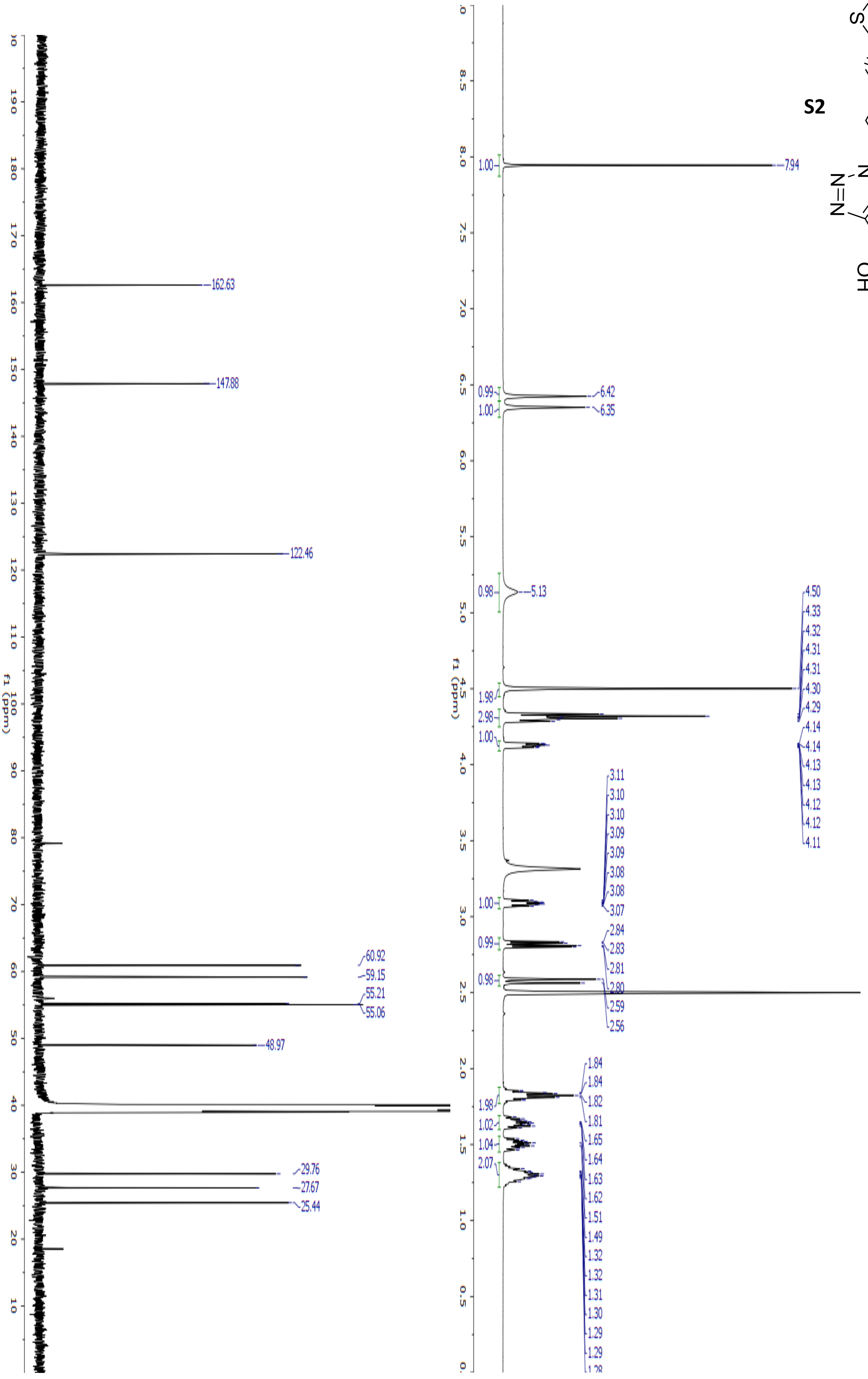

f1 (ppm)
162.63
147.88
122.46
60.92
59.15
55.21
55.06
48.97
29.76
27.67
25.44
7.94
6.42
6.35
5.13
4.50
4.33
4.32
4.31
4.31
4.30
4.29
4.14
4.14
4.13
4.13
4.12
4.12
4.11
3.11
3.10
3.10
3.09
3.09
3.08
3.08
3.07
2.84
2.83
2.81
2.80
2.59
2.56
1.84
1.84
1.82
1.81
1.65
1.64
1.63
1.62
1.51
1.49
1.32
1.32
1.31
1.30
1.29
1.29
1.28

S2

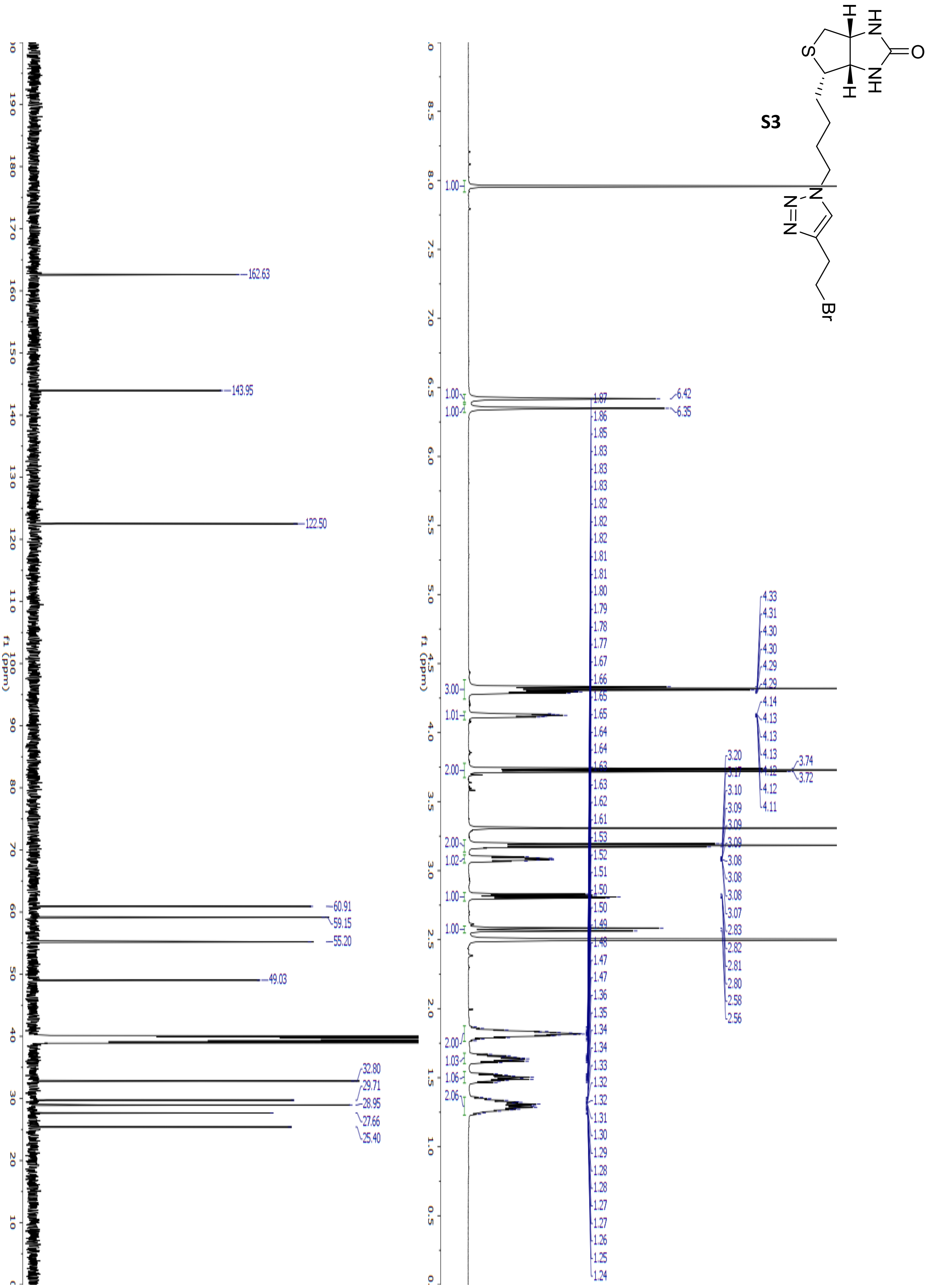

S3

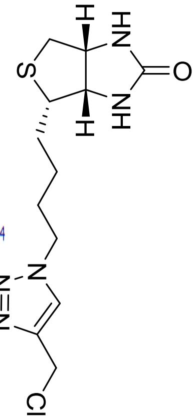

3b

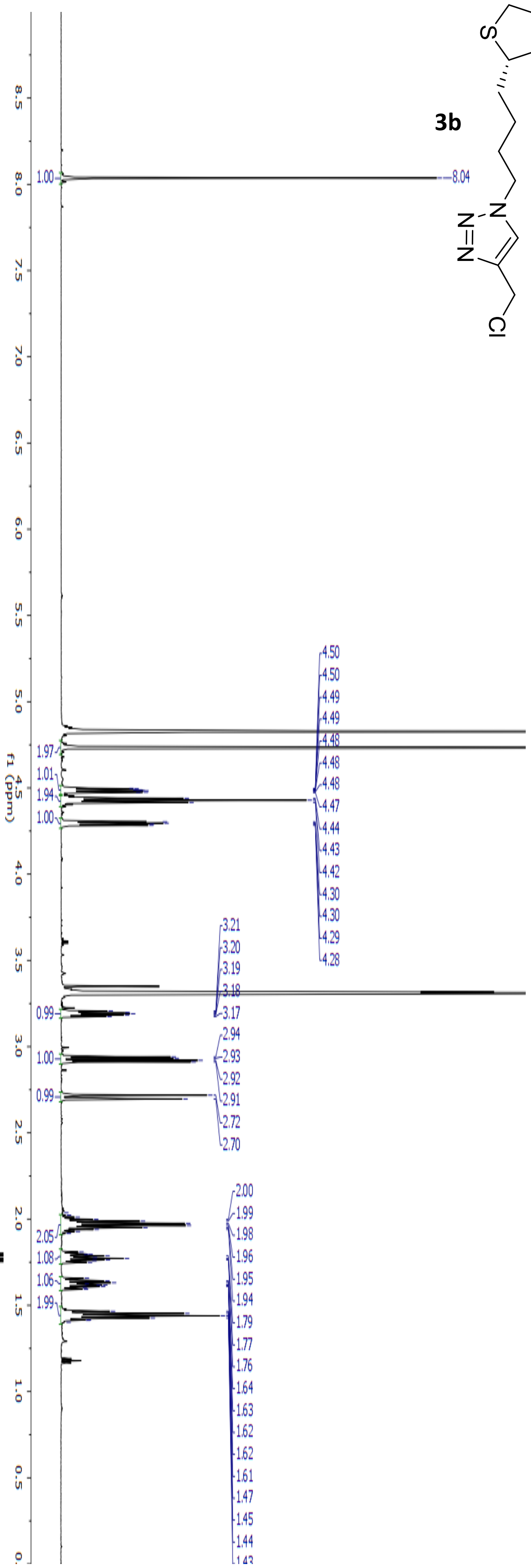

8.04
1.00
4.50
4.50
4.49
4.49
4.48
4.48
4.48
4.47
4.44
4.43
4.42
4.30
4.30
4.29
4.28
1.97
1.01
1.94
1.00
3.21
3.20
3.19
3.18
3.17
0.99
2.94
2.93
2.92
2.91
2.72
2.70
1.00
0.99
2.00
1.99
1.98
1.96
1.95
1.94
1.79
1.77
1.76
1.64
1.63
1.62
1.62
1.61
1.47
1.45
1.44
1.43
2.05
1.08
1.06
1.99
f1 (ppm)

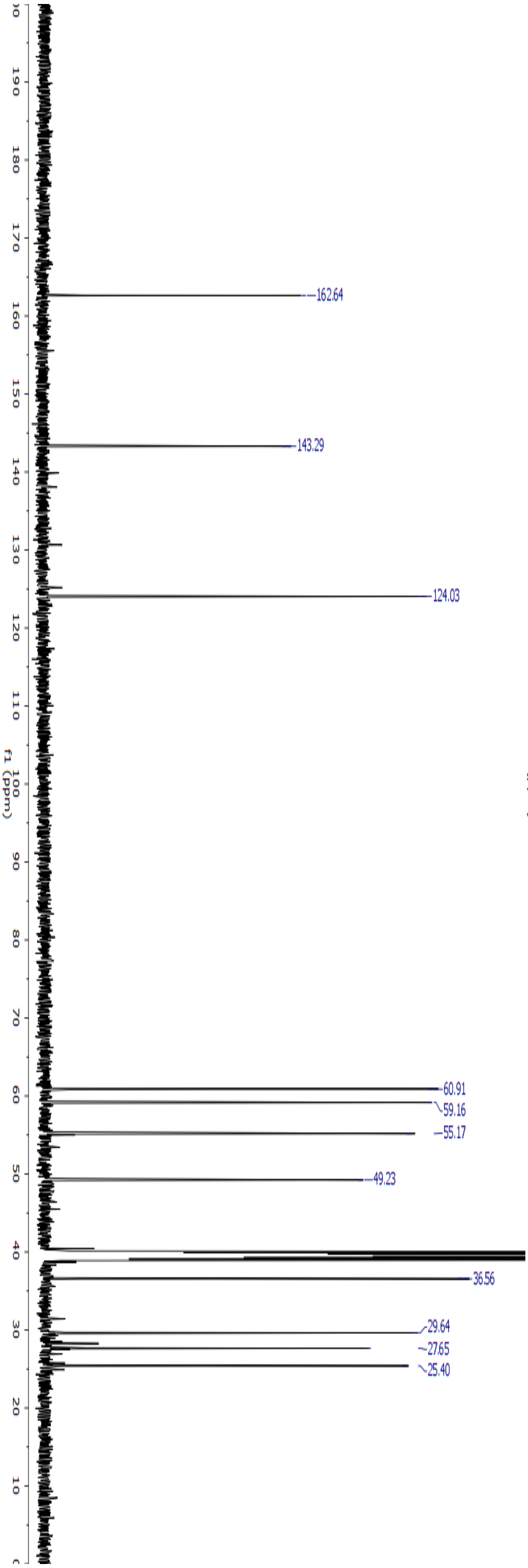

162.64
143.29
124.03
60.91
59.16
55.17
49.23
36.56
29.64
27.65
25.40
f1 (ppm)

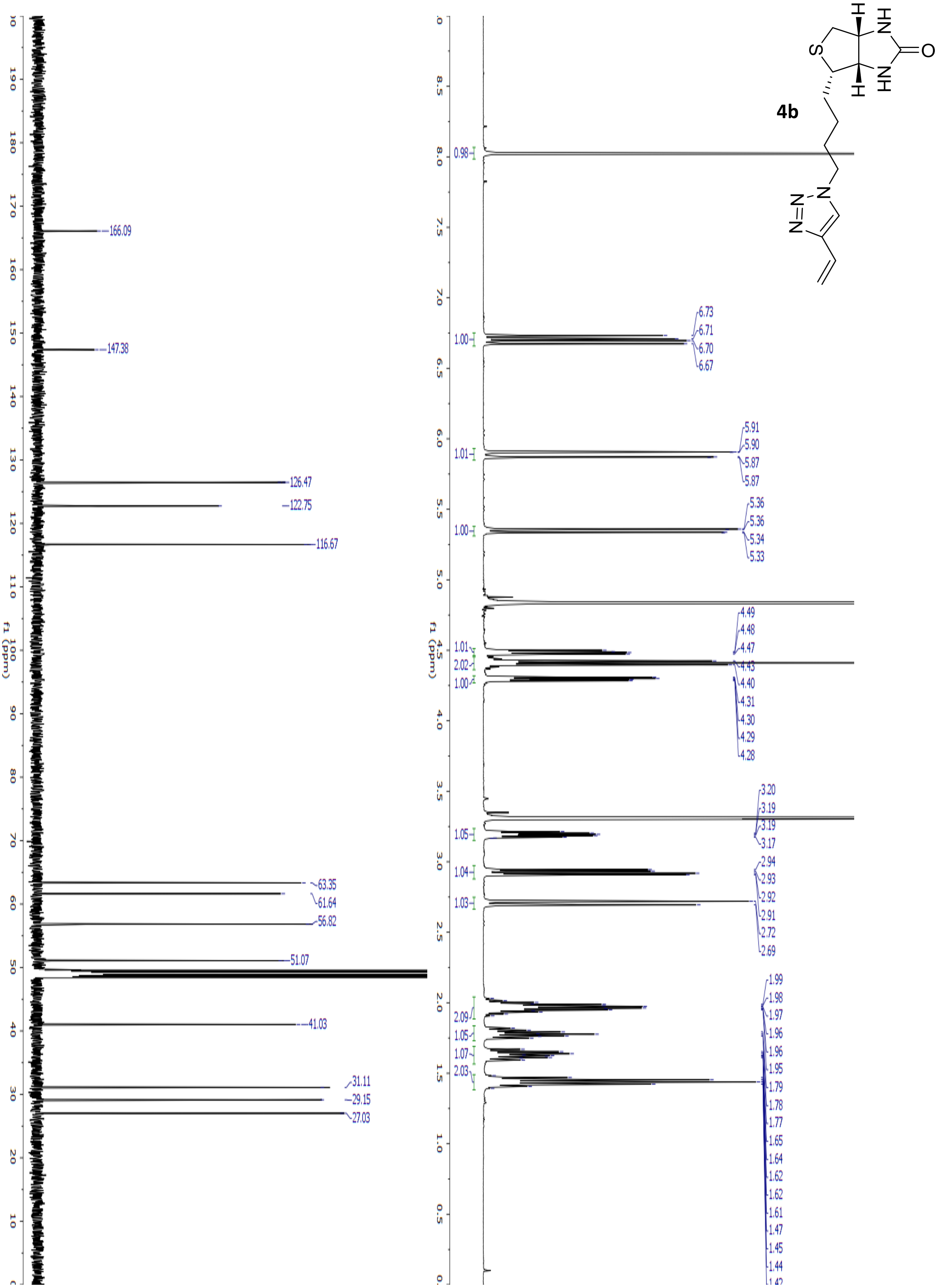
4b
f1 (ppm)
f1 (ppm)
